# Notification Strategy and Predictors of Outcome in Stroke Ineligible for Reperfusion Therapies

**DOI:** 10.3389/fneur.2019.01060

**Published:** 2019-10-10

**Authors:** Ágnes Mirolovics, Magdolna Bokor, Balázs Dobi, Judit Zsuga, Dániel Bereczki

**Affiliations:** ^1^János Szentágothai Doctoral School of Neurosciences, Semmelweis University, Budapest, Hungary; ^2^Department of Neurology, National Institute of Psychiatry and Addictions Nyíro Gyula, Budapest, Hungary; ^3^Department of Probability Theory and Statistics, Eötvös Loránd University, Budapest, Hungary; ^4^Department of Health Systems Management and Quality Management in Health Care, Faculty of Public Health, University of Debrecen, Debrecen, Hungary; ^5^Department of Neurology, Semmelweis University, Budapest, Hungary; ^6^MTA-SE Neuroepidemiological Research Group, Budapest, Hungary

**Keywords:** stroke, emergency medical service notification, disability, survival, lack of reperfusion

## Abstract

**Background, Objective:** At least 70% of all stroke patients are ineligible for recanalization therapy. We identified predictors of outcome among these patients, with special focus on notification of emergency medical services (EMS).

**Methods:** We prospectively collected data of 250 consecutive patients with acute cerebrovascular diseases ineligible for recanalization therapy. Initial notification strategy and outcome were analyzed by regression models.

**Results:** EMS notification rate was 55, 41, and 21% in patients with <6, 6–24, and >24 h stroke-to-door time. Atrial fibrillation (AF; OR = 2.66, 95% CI: 1.19–5.96), stroke severity (National Institutes of Health Stroke Scale score, NIHSS; OR = 1.12, 95% CI: 1.02–1.23), history of any psychiatric disease (OR = 2.2, 95% CI: 0.98–4.97), aphasia (OR = 1.99, 95% CI: 0.99–3.98), and residence type were predictors of EMS notification. Disability (modified Rankin Scale score [mRS]) both at discharge and at 1 year was associated with age, admission NIHSS score, type of cerebrovascular disorder, and pre-stroke mRS at discharge and discharge mRS at follow-up. Age (HR = 1.05, 95% CI: 1.02–1.08) and NIHSS (HR = 1.16, 95% CI: 1.12–1.21) had a significant effect on the relative hazard of death.

**Conclusions:** EMS notification is influenced by AF, stroke severity, psychiatric disease, aphasia, and residence type. Early disability depends on age, the type and severity of the stroke, and pre-stroke mRS. Predictors of disability at 1 year after stroke are age, stoke severity, mRS at discharge, and recurrent ischemic stroke. Higher NIHSS and older age are associated with higher case fatality. In patients ineligible for recanalization, EMS notification had no significant effect on outcome, regarding both disability and survival.

## Introduction

Intravenous thrombolysis has become the routine practice for treating patients with acute ischemic stroke. Current guidelines recommend the emergency medical services (EMS) to bypass hospitals that are unable to perform hyperacute interventions for patients who are candidates for intravenous or intra-arterial recanalization treatments (1). In recent decades, there was a considerable increase in the rate of intravenous thrombolysis (IVT) worldwide, and with proper organization of services, the rate of IVT can be increased up to 25% (2). Even if the thrombolysis rate is as high as 35% (3), two-thirds of patients with acute stroke do not get reperfusion treatment.

Due to limited capacity of primary and comprehensive stroke centers, patients ineligible for IVT or mechanical thrombectomy (MT) are either transported further to units with no facilities for reperfusion therapies, or primarily admitted to such units if it is obvious at the initial EMS examination that neither IVT nor MT can be performed. Limited data are available for this large volume of stroke patients.

An area of interest are the factors influencing notification and stroke-to-door time and their effect on prognosis. Matsuo et al. reported that early hospital arrival within 6 h of stroke onset was associated with neurological improvement during hospitalization and good functional outcome after 3 months in patients with acute ischemic stroke. These associations existed even in patients without reperfusion treatment and in those with minor stroke and were independent of age, sex, stroke severity, and stroke subtype (4).

Barsan et al. found that early hospital arrival after stroke was greatly influenced by the type of first medical contact and, to a lesser degree, by the patient's location at the time of the stroke and the time of the day at which the stroke occurred. Hospital arrival was fastest in patients using EMS as their first medical contact vs. their personal physician or a study hospital (5). Williams et al. found that even patients who are aware that they are having a stroke, often delay notification as they deem their symptoms as “not serious” (6).

A Korean study found that the rate of correct diagnosis for stroke was much higher and the real transfer time was much faster in patients with an EMS thrombolysis prenotification than in those without one. Also, the door-to-imaging and door-to-needle times were significantly shorter for patients with EMS prehospital notification than for those without it (7).

In a consecutive set of stroke patients ineligible for IVT or MT, we evaluate their notification strategy when experiencing stroke symptoms, and we also analyze predictors of short- and long-term outcome.

### Primary and Secondary Endpoints

We hypothesized that the strategy of initial notification of the emergency services benefits functional outcome even in patients who are ineligible for recanalization therapies. Therefore, we examined which factors may affect EMS call as an initial notification strategy.

Further, we evaluated the effect of EMS call as an initial notification strategy on disability (mRS) at hospital discharge, and on survival and disability after 1 year in patients who are ineligible for recanalization therapies. In the primary analysis, EMS notification was handled as a binary variable (called EMS vs. did not call EMS). In a secondary analysis, patients with EMS call and <6 h stroke-to-door time were compared to a combined group of those with EMS call with over 6 h stroke-to-door-time and those who had not called the EMS at all.

## Methods

### Catchment Area and Admission Policy

Data of 250 consecutive patients admitted for acute cerebrovascular disease between February 2013 and April 2014 to the Department of Neurology of the Nyíro Gyula National Institute of Psychiatry and Addictions, Budapest, Hungary, were prospectively collected. The department has no onsite facilities for IVT or MT. The department is responsible for the neurological care of the 13th district of Budapest, as well as the citizens of two other Hungarian towns: Pilisvörösvár and Csobánka. These areas cover approximately 133,000 people.

CT scan is available for 24 h only 4 days per week at the department, during the rest of the week, it is available during work hours (8 h a day). When CT cannot be performed locally, it is performed in another hospital, which is available in 10 min. The primary stroke center in our district that is responsible for treating potentially eligible patients for IVT is the nearby Hungarian Army Medical Hospital (HAMH). Patients potentially eligible for recanalization therapy are primarily transported to HAMH from the catchment area, and in the case of contraindications for intervention, the patients are transferred to our unit. The rest of the patients were admitted directly to our hospital through the EMS or the GP system.

We enrolled all inpatient cases with acute cerebrovascular disorder who were admitted to our department during the 13 month period of our study except for those who were admitted after IVT or MT treatment at a primary stroke center. Of the 250 patients included in the study, there were 187 cases of ischemic stroke, 16 cases of intracerebral hemorrhage, 1 case of subarachnoid hemorrhage, and 46 cases of transient ischemic attack. Of the 250 patients, 89 (35.3%) were originally admitted to the HAMH and, being ineligible for IVT or MT, were transferred further to the department of the study, according to the regional patient admission rules.

### Data Collection

We collected information using healthcare data and a *structured questionnaire* within the first week of hospitalization. The same neurologist performed both the patient examination and data collection. We recorded stroke severity on admission by the National Institutes of Health Stroke Scale (NIHSS) (8), as well as the affected brain hemisphere, presence of speech disturbance, and routine laboratory values. We also recorded the presence of hypertension, diabetes mellitus, atrial fibrillation (AF), heart disease, other arrhythmia, peripheral vascular disease, psychiatric disease, and liver and lung disease. We recorded data on alcohol consumption, smoking, regular medications, type of the earlier stroke, treatment of AF if present, the pre-stroke modified Rankin scale score (mRS), the CHA_2_DS_2_VASc score (9), the HAS-BLED score (10), admission brain CT scan results (no change, ischemic or hemorrhagic lesion, or subarachnoid hemorrhage), blood pressure, and heart rate.

Regarding sociodemographic data, we recorded the type of residence (stand-alone house, apartment in building made of brick, panel apartment houses built of concrete—i.e., the main urban housing built in the Soviet era, also called Larsen-Nielsen-type building—and other types: retirement home, homeless shelter), marital state, education, profession, property ownership, monthly income per capita, and number of children.

We also recorded the notification strategy, i.e., *who did the patient notify first after experiencing stroke symptoms*: called the EMS, called the general practitioner (GP) on-call medical service, visited the GP, notified a relative or an acquaintance, waited and took medication available at home, or waited without action. In patients with lack of communication (aphasia or severe general condition), we recorded the measurable data only and gained information about the initial notification strategy from family or medical documentation.

Additional information relating to hospital stay and discharge were also recorded, such as the TOAST classification (11) of the stroke, the findings of carotid duplex scan and of echocardiography, scores on two depression tests [Beck Depression Inventory (12), Center for Epidemiologic Studies Depression Scale (CES-D scale) (13)], medication use on the ward (anticoagulants, antihypertensives, antidiabetics, statins), and condition at discharge (survival status and mRS score).

A 1 year follow-up assessment with a mean of 13.7 months from the stroke event was performed via telephone interview. Survival status, new stroke event, and mRS were recorded.

### Compliance With Ethical Standards

All procedures performed in this study involving human participants were in accordance with the ethical standards of the Ethics Committee of Semmelweis University, Budapest, Hungary, and with the 1964 Helsinki declaration and its later amendments or comparable ethical standards.

### Ethical Approval and Informed Consent

The study was approved by the Ethics Committee of Semmelweis University, Budapest, Hungary (No: TUKEB 8/2013), and written consent was obtained from the patients.

### Data Analysis

Logistic regression was used for the analysis of *notification strategy*. The effect of sex, age, stroke type, sociodemographic factors, the presence of AF, the admission NIHSS score, comorbidities, and health consciousness variables were considered together in the model.

Early and late disability and case fatality were the clinical outcomes of interest. Several variables were considered during the evaluation of the *predictors of outcome*. The potential covariates were chosen according to their viability in the given model, judged by medical professionals. The effects of the variables were first considered together only with sex, age, and admission NIHSS, and later together with other possible predictors. Only those variables, which had a significant effect at the 0.1 level, were selected for the more complex models. After the inclusion of all potential covariates in the models, bidirectional stepwise regression procedure based on the Akaike information criterion was used for model selection. For a single variable, this algorithm would correspond to using a critical *p*-value of 0.157. This stepwise algorithm was used only as a supporting tool during model selection, as initial variables in the models were manually added according to their viability, and resulting models were also inspected and corrected afterwards. Sex and age were kept in all models as control variables and the effect of early EMS notification (within 6 h) was also checked in all of the final models.

Predictors of the *discharge and follow-up disabilities (mRS)* were analyzed using ordinal logistic regression. The effect of age, sex, admission NIHSS, EMS notification, the presence of AF, admission and follow-up stroke type, sociodemographic factors, disability before stroke (mRS), comorbidities, medications received in the hospital, stroke-to-door time, health consciousness variables, and—during the analysis of follow-up mRS—the discharge mRS were considered.

*Survival* was analyzed using Kaplan–Meier survival analysis and the Cox proportional hazards model. In the Cox regression model, sex, age, admission NIHSS, EMS notification, stroke type, sociodemographic factors, comorbidities, medications received in the hospital, stroke-to-door time, and health consciousness variables were considered as covariates.

Goodness of fit was tested using statistical and visual tools. These were the Hosmer–Lemeshow test and separation plot (14) for logistic regression. For the ordinal models, the Hosmer–Lemeshow test was also used, and the predicted values were plotted for checking the proportional odds assumption. Chi-square test and Schoenfeld residual plots were used for the Cox proportional hazards model. R statistical software, version 3.5.1, with packages survival, survminer, generalhoslem, MASS, separationplot, ggplot2, and Hmisc were used during data analysis.

## Results

### Predictors of the Initial Notification Strategy

Trichotomizing stroke-to-door time to early (<6 h, *n* = 58; 24%), intermediate (6–24 h, *n* = 92; 38%), and late (over 24 h, *n* = 92; 38%) admission, we found that with the increase of stroke-to-door time, the proportion of patients with EMS notification as an initial strategy decreased. Of the 58 patients admitted within 6 h, 32 (55%) were admitted by EMS. In the stroke-to-door time range of 6–24 h, EMS transport rate was 41%, whereas among those who reached the hospital more than 24 h after stroke onset, only 22% used the EMS. For those eight patients who were admitted from distant hospital wards, stroke-to-door time was not relevant in this evaluation.

The reasons for exclusions from reperfusion treatment among the 32 patients delivered by EMS with a <6 h stroke-to-door time were hemorrhagic stroke, high or low NIHSS, age, TIA, high serum glucose level, dementia, recent surgery, low platelet count, high INR, high blood pressure, and uncertain stroke-to-door time (within 4.5–6 h).

Table 1 shows initial stroke severity (NIHSS score) stratified by the notification strategy. NIHSS was the highest among those who initially called the EMS (Kruskal–Wallis rank sum test, *p* < 0.001).

**Table 1:** NIHSS by initial notification strategy.

**Who was first notified?**	**Number of patients**	**NIHSS mean**	**NIHSS standard deviation**	**NIHSS first quartile**	**NIHSS median**	**NIHSS third quartile**
EMS	91	7.93	6.94	2.50	6.00	13.00
GP on call service	47	3.79	4.55	1.00	2.00	4.50
Visited the GP	46	3.04	3.13	0.25	2.00	5.00
Relative, friend	39	3.58	3.49	0.25	3.00	6.00
Waited, did nothing	19	3.05	2.61	0.50	2.00	6.00
Delayed transferred from a hospital other than HAMH	8	10.88	5.30	9.75	10.50	14.50

*NIHSS, National Institutes of Health Stroke Scale; EMS, Emergency Medical Service; GP, general practitioner; HAMH, Hungarian Army Medical Hospital*.

In the analysis of the notification strategy, 201 patients were included, as the analysis was restricted to cases where information on all analyzed predictors were available with no missing values. Logistic regression was used for the analysis of notification strategy (Table 2). According to the Hosmer–Lemeshow goodness-of-fit test (*p* = 0.965) and the separation plot, a reasonably good fit was achieved. The presence of AF (OR = 2.66, 95% CI: [1.19–5.96], *p* = 0.017), initial stroke severity (NIHSS, OR = 1.12, 95% CI: [1.02–1.23], *p* = 0.022), the history of psychiatric disease (OR = 2.20, 95% CI: [0.98–4.97], *p* = 0.057), the presence of aphasia (OR = 1.99, 95% CI: [0.99–3.98], *p* = 0.052), and the residence type were predictors of EMS notification. The residence type was analyzed with the stand-alone house as reference category (*n* = 28). Compared to this residence type, all other types of housing showed increased odds in EMS notification: apartment made of brick (*n* = 126; OR = 10.96, 95% CI: [1.35–88.67], *p* = 0.025), panel apartment houses built of concrete (main urban housing built in the Soviet era) (*n* = 39, OR = 18.79, 95% CI: [2.16–163.75], *p* = 0.008), and other types (*n* = 10, OR = 47.49, 95% CI: [3.74–603.15], *p* = 0.003). The effects of sex and age were not significant predictors of EMS notification. Predictors of EMS call with a stroke-to-door time of <6 h were evaluated in a separate analysis, and initial stroke severity (NIHSS, OR = 1.11, 95% CI: [1.04–1.18], *p* < 0.001) and the history of psychiatric disease (OR = 2.79, 95% CI: [1.14–6.82], *p* = 0.025) were the significant predictors of EMS notification with early hospital arrival.

**Table 2:** Logistic regression model for the notification strategy (EMS call).

**Reference categories**	**Variables (*n* = 201)**	**Odds ratio**	**Lower 95% CI**	**Upper 95% CI**	***p*-value**
Male	Sex—female	1.07	0.53	2.16	0.847
	Age	0.98	0.95	1.01	0.141
	Admission NIHSS	1.12	1.02	1.23	0.022
Not present	Atrial fibrillation—present	2.66	1.19	5.96	0.017
Not present	Psychiatric disease—present	2.20	0.98	4.97	0.057
Not present	Aphasia—present	1.99	0.99	3.98	0.052
Stand-alone house	Apartment made of brick	10.96	1.35	88.67	0.025
	Panel apartment houses built of concrete (main urban housing built in the soviet era)	18.79	2.16	163.75	0.008
	Other type	47.49	3.74	603.15	0.003

*CI, Confidence interval; NIHSS, National Institutes of Health Stroke Scale*.

### Predictors of Disability (mRS) at Discharge and 1 Year After Stroke

The discharge and follow-up mRS were analyzed using ordinal logistic regression. Only surviving patients were included in the analysis—those who had mRS below six. This was justified by a better fit, and the fact that survival was analyzed separately. According to the Hosmer–Lemeshow test for ordinal models, the goodness of fits of the models were acceptable (discharge mRS: *p* = 0.822, follow-up mRS: *p* = 0.586). Graphical checking of the proportional odds assumption indicated that the assumption may not hold in every case; thus, the results should be interpreted and used with caution.

Predictors of mRS at hospital discharge and at 1 year follow-up can be seen in Table 3. Disability at discharge (mRS) is associated with age, admission NIHSS, findings at acute imaging, and disability before stroke.

**Table 3:** Ordinal logistic regression models for discharge and follow-up mRS.

	**Reference categories**	**Variables**	**Odds ratio**	**Lower 95% CI**	**Upper 95% CI**	***p*-value**
Discharge mRS, *n* = 218	Male	Sex—female	0.66	0.36	1.23	0.195
		Age	1.03	1.01	1.06	0.010
		Admission NIHSS	1.55	1.41	1.73	<0.001
	Neuroimaging result: No deviation or only non-acute ischemia on the CT	Stroke type—new ischemic	3.68	1.70	8.29	0.001
		Stroke type—hemorrhagic/subarachnoid hemorrhage	14.27	3.25	64.56	<0.001
		TIA	0.40	0.11	1.30	0.136
	Pre-stroke mRS = 0–1	Pre-stroke mRS >1	7.47	3.13	18.15	<0.001
	No EMS notification within 6 h	EMS notification within 6 h	0.69	0.28	1.64	0.401
Follow-up mRS, *n* = 150	Male	Sex—female	1.01	0.43	2.38	0.982
		Age	1.05	1.02	1.09	0.006
		Admission NIHSS	1.19	1.04	1.37	0.014
	Pre-stroke mRS = 0–1	Pre-stroke mRS >1	1.77	0.52	6.20	0.365
	Discharge mRS = 0	Discharge mRS = 1	7.68	2.53	25.15	<0.001
		Discharge mRS = 2	27.88	7.17	117.22	<0.001
		Discharge mRS = 3	172.11	27.17	1,255.56	<0.001
		Discharge mRS = 4	255.78	46.64	1,605.84	<0.001
		Discharge mRS = 5	1,772.87	163.14	28,357.84	<0.001
	Recurrent cerebrovascular disease type: No new cerebrovascular disease	New stroke type—ischemic	15.18	4.00	60.91	<0.001
		New TIA	2.67	0.11	26.13	0.445
	No EMS notification within 6 h	EMS notification within 6 h	1.34	0.40	4.46	0.631

*CI, Confidence interval; mRS, modified Rankin Score; NIHSS, National Institutes of Health Stroke Scale; TIA, transient ischemic attack*.

Predictors of disability with significant effect 1 year after stroke included age, admission NIHSS, discharge mRS, and recurrent ischemic stroke during follow-up. We found weak evidence of the effect of admission NIHSS and discharge mRS. EMS notification status and the presence of AF were considered during modeling, but neither of them was associated with short-term or long-term disability.

Early disability and late disability were not affected by EMS call in a separate analysis where EMS call with <6 h stroke-to-door time was compared to combined group of those with EMS call with over 6 h stroke-to-door time and those who had not called the EMS at all. In this analysis, EMS call was not associated with either discharge mRS (OR = 0.69, 95% CI: [0.28–1.64], *p* = 0.401) or follow-up mRS: (OR = 1.34, 95% CI: [0.40–4.46], *p* = 0.631).

### Survival in the First Year After Stroke

Twelve months after the acute event, the probability of survival was 70% (95% CI: [65%−77%]) according to the Kaplan–Meier survival analysis. Patients who did not die were censored after the 12 month follow-up.

Survival was also analyzed by Cox proportional hazards model (Table 4). According to the global chi-squared test for the proportional hazards assumption (*p* = 0.250) and the Schoenfeld residual plots, the fit was reasonably good (the plots indicated some deviation from the proportional hazards assumption though). Significant predictors of fatal outcome were older age and higher admission NIHSS, whereas administration of statin and antiplatelet medication decreased the relative hazard of death. EMS notification even with a <6 h stroke-to-door time had no significant effect on survival in patients ineligible for reperfusion treatment (HR = 0.66, 95% CI: [0.33–1.31], *p* = 0.233).

**Table 4:** Cox proportional hazards model for death.

**Reference categories**	**Variables**	**Hazard ratios**	**Lower 95% CI**	**Upper 95% CI**	***p*-value**
	***n* = 227, number of events = 63**				
Male	Sex—female	0.95	0.51	1.79	0.881
	Age	1.05	1.02	1.08	<0.001
	Admission NIHSS	1.16	1.12	1.21	<0.001
Not given	Statin—given in hospital	0.25	0.10	0.64	0.004
Not given	Antiplatelet drugs—given in hospital	0.48	0.29	0.81	0.006
No EMS notification within 6 h	EMS notification within 6 h	0.66	0.33	1.31	0.233

*CI, Confidence interval; NIHSS, National Institutes of Health Stroke Scale*.

## Discussion

We performed a study on patients with acute cerebrovascular diseases who are ineligible for reperfusion therapies. We assumed that even in these patients, EMS call as the initial notification strategy will improve outcome; therefore, we focused on factors influencing EMS call after stroke and analyzed the effect of initial EMS notification on functional outcome (mRS) and survival. We found that EMS notification is a more frequent initial strategy in those with more severe stroke, who have AF, history of psychiatric disorder or aphasia, and less frequent among those residing in stand-alone houses. Major predictors of early and late disability and 1 year case fatality were older age and more severe initial stroke. Disability at discharge (mRS) is associated with age, admission NIHSS, acute ischemic or hemorrhagic lesion on neuroimaging, and pre-stroke mRS. Disability at 1 year is predicted by disability at discharge and stroke recurrence during the 1 year follow-up. In patients ineligible for reperfusion treatment, the initial notification strategy of EMS call had no effect on early and late disability and 1 year case fatality even in those with a <6 h stroke-to-door time.

Currently at least two-thirds of stroke patients do not receive reperfusion therapy (IVT/MT). Still, there is little attention given to this patient group. Our 13 month research was conducted on 250 consecutive patients with stroke or TIA admitted to a hospital lacking facilities for recanalization therapies. Acute stroke cases from the catchment area who are potentially eligible for IVT or MT are primarily admitted to a nearby hospital (HAMH) with a stroke center. The site of the current study admits patients ineligible for reperfusion therapy either primarily by the EMS or after initial evaluation by the stroke center of the nearby hospital. Due to this admission policy, on the site of the study, it was not possible to make a comparison with those receiving reperfusion treatment. In addition to the notification strategy, our survey included clinical features of the acute stroke, risk factors, and socioeconomic features, which were evaluated by multivariable statistical methods.

We found that more severe stroke, aphasia, the presence of psychiatric disease, and certain housing conditions increased the odds of EMS notification. The effect of the type of residence can be explained by the effect of urbanization and socio-economic status. Furthermore, AF was also independently associated with initial EMS call. Schroeder et al. found that older individuals were more likely to use EMS, and having somebody other than the patient first identify that there was a problem was also a strong predictor of EMS call (15). Our model showed no association between age and EMS notification.

We also examined what factors influence functional outcome at discharge and 1 year later in stroke patients ineligible for reperfusion therapy. Disability at discharge (mRS) is associated with age, admission NIHSS, acute ischemic or hemorrhagic lesion on neuroimaging, and pre-stroke mRS. Predictors of disability with significant effect 1 year after stroke include age, admission NIHSS, discharge mRS, and stroke recurrence. These results are in line with a systematic review of articles published from January 2008 to May 2018, which found that the severity of the initial stroke is a primary determinant of the clinical outcome. The NIHSS and the mRS appear to be predictive tools of the functionality of the patient with ischemic stroke, especially in the acute phase (16). Also, an Italian study found that stroke severity and advanced age, together with the need of urinary catheter, oxygen administration, and persistence of upper limb paralysis, allow a simple and accurate prediction of dependency or death after ischemic stroke (17). Immediate EMS notification after stroke and the presence of AF were also considered during modeling but neither of them was associated with short-term or long-term disability. A study on 721 patients admitted consecutively for TIA or stroke to 18 Spanish hospitals found that patients with worse neurological condition presented earlier, but the case fatality was not modified by earlier or late presentation. The delays before the patient was seen by the first physician or the emergency department and before hospitalization were not independently related to clinical outcome (18). Similarly to our findings, in 178 patients ineligible for IVT or MT, 3 month outcome was significantly associated with admission NIHSS, age, and pre-stroke mRS, and marginally significantly with time to presentation (19). Severe disability or death 1 month after stroke in patients ineligible for recanalization therapy was associated with age, AF, and pre-stroke disability, and longer than 4.5 h to presentation (20).

Twelve months after the primary data assessment, more than two thirds of our patients were alive. Age and higher NIHSS at admission increased, whereas administration of statin and antiplatelet medications decreased the relative hazard of death significantly.

For our patients who are ineligible for recanalization treatments, the effect of EMS notification on both short- and long-term outcome, regarding both disability and survival rate, is negligible compared to the severity of the stroke. Initial EMS notification, however, does not necessarily mean arrival to a stroke center within the therapeutic time window: In our study, over 40% of those with a stroke-to-door time of 6–24 h and 22% of those admitted more than 24 h after stroke had EMS transport to the hospital.

Although initial EMS notification did not improve functional outcome in patients ineligible for reperfusion therapies, emergent EMS notification in stroke should be emphasized in public campaigns to increase the rate of timely admission of those who are eligible for recanalization treatments, thus decreasing death and dependency in stroke survivors. Early recognition by paramedics is associated with higher rates of thrombolytic therapy (21). The door-to-needle time was significantly reduced during a period with pre-hospital notification compared with a period without pre-hospital notification found by Kim et al. (22).

### Study Limitations

One of the limitations of our study is the relatively low number of cases. Regarding some variables—e.g., residence type—this results in wide confidence intervals. Although notification strategy was known in all patients, altogether 201 patients were included in the multivariate analysis of the notification strategy, as the analysis was restricted only to cases where information on all analyzed predictors were available. Although 4 years has elapsed from the end of the follow-up, as no major changes have been performed in stroke care services for patients ineligible for reperfusion treatment, we assume that our conclusions are still valid. Further, although we included consecutive patients in a single center, a selection bias may be present, as we cannot tell the number and features of patients who were admitted to other hospitals from the catchment area. Finally, as our data show, initial EMS notification may be associated with various stroke-to-door times; thus, no unequivocal conclusions can be made on the effect of time to presentation and outcome.

## Conclusions

EMS notification strategy is influenced by AF, stroke severity, psychiatric disease, aphasia, and residence type. Early disability depends on age, the type and severity of the stroke, and the pre-stroke mRS. Predictors of disability at 1 year after stroke are age, stoke severity, mRS at discharge, and recurrent ischemic stroke. EMS notification strategy was not independently associated with disability or survival in acute stroke patients ineligible for reperfusion therapies.

## Data Availability Statement

The datasets generated for this study are available on request to the corresponding author.

## Ethics Statement

The study was approved by the Ethics Committee of Semmelweis University, Budapest, Hungary (No: TUKEB 8/2013). The patients/participants provided their written informed consent to participate in this study. Written informed consent was obtained from the individual(s) for the publication of any potentially identifiable images or data included in this article.

## Author Contributions

DB designed and supervised the study. ÁM performed patient examination and data collection. BD performed statistical analysis of the data. ÁM and BD drafted the manuscript. JZ and MB edited the manuscript for important intellectual and clinical content. All authors read and approved the final version of the manuscript.

### Conflict of Interest

The authors declare that the research was conducted in the absence of any commercial or financial relationships that could be construed as a potential conflict of interest.
